# TNFAIP8 protein functions as a tumor suppressor in inflammation-associated colorectal tumorigenesis

**DOI:** 10.1038/s41419-022-04769-x

**Published:** 2022-04-06

**Authors:** Yunwei Lou, Xueqin Tian, Chen Sun, Miaomiao Song, Meijuan Han, Yuxin Zhao, Yaru Song, Xiangfeng Song, Wen Zhang, Youhai H. Chen, Hui Wang

**Affiliations:** 1 Henan Key Laboratory of Immunology and Targeted Drugs, Xinxiang Medical University, 453003 Xinxiang, Henan China; 2 Henan Collaborative Innovation Center of Molecular Diagnosis and Laboratory Medicine, School of Laboratory Medicine, Xinxiang Medical University, 453003 Xinxiang, Henan China; 3 Department of Neurosurgery, The First Affiliated Hospital of Zhengzhou University, 450052 Zhengzhou, Henan China; 4 Department of Immunology, Xinxiang Medical University, 453003 Xinxiang, Henan China; 5 Morphologic Center of College of Basic Medicine, Xinjiang Medical University, 830011 Urumqi, Xinjiang China; 6 Department of Pulmonary Medicine, The Affiliated Renmin Hospital of Xinxiang Medical University, 453100 Xinxiang, Henan China; 7 School of Pharmaceutical Sciences, Shenzhen Institute of Advanced Technology (SIAT), Chinese Academy of Sciences, 510855 Shenzhen, China

**Keywords:** Cancer microenvironment, Chronic inflammation

## Abstract

Tumor necrosis factor-α-induced protein 8 (TNFAIP8 or TIPE) is a member of the TNFAIP8 family. While TIPE was broadly considered to be pro-cancerous, its precise roles in carcinogenesis especially those of the intestinal tract are not clear. Here, we show that genetic deletion of TIPE in mice exacerbated chemical-induced colitis and colitis-associated colon cancer. Loss of TIPE exacerbated inflammatory responses and inflammation-associated dysbiosis, leading to the activation of NF-κB and STAT3, and it also accelerated dysplasia, DNA damage and proliferation of intestinal epithelial cells. We further show that colon microbiota were essential for increased tumor growth and progression in *Tipe*^*−/−*^ mice. The tumor suppressive function of TIPE originated primarily from the non-hematopoietic compartment. Importantly, TIPE was downregulated in human colorectal cancers, and patients with low levels of *Tipe* mRNA were associated with reduced survival. These results indicate that TIPE serves as an important modulator of colitis and colitis-associated colon cancer.

## Introduction

Colorectal cancer (CRC) is the third most prevalent cancer and one of the major killers of cancer patients in the world, resulting in death of more than half a million every year [1]. Patients with inflammatory bowel disease (IBD) increase the risk of developing colitis-associated colon cancer (CAC). Recent studies have demonstrated that host-microbial homeostasis involves appropriate immune regulation within the intestinal mucosa to maintain a healthy intestine while preventing uncontrolled immune responses [2]. Although the epithelium and microbiota components contribute to colitis and CAC in a context-dependent manner, it is not completely understood how the damaged epithelium is effectively repaired to rebuild intestinal homeostasis to avoid inflammation-mediated pathogenesis. Thus, exploring the relationship between intestinal inflammation and CRC progress may offer new therapies to prevent CAC.

The mammalian gastrointestinal tract is colonized with more than 100 trillion microbes. These gut microbiota participate in shaping both innate and adaptive immune response and regulating the pathogenesis of intestinal diseases, including IBD and CRC [3]. Accumulating evidences have shown an association between CRC and changes of the overall community structure of the gut microbiota, referred to as “dysbiosis” [4]. Human studies demonstrated that the gut microbiota related to CRC were different compared with the gut microbiota of healthy individuals, which was characterized by an outgrowth of certain procarcinogenic taxa (such as *Fusobacterium nucleatum*, *Escherichia coli*, *Bacteroides fragilis*, *Enterococcus faecalis* and others) [5–7]. Moreover, transfer of stool from patients with CRC to mice enhanced intestinal cell proliferation in germ-free mice and promoted tumor development in conventional mice given azoxymethane to induce colon neoplasia [8]. These data provide direct evidence for the functional importance of the microbial community on carcinogenesis, and pinpoint a potential core set of microbiota that might be carcinogenic. In particular, specific members of the Enterobacteriaceae family expand during bouts of inflammation, which in turn exacerbates epithelial cell damage, wound repair responses and intestinal prolonged inflammation [9].

TNFAIP8 (Tumor necrosis factor-α-induced protein 8, also known as TIPE), is the first identified member of TNFAIP8 family of proteins [10–12]. TIPE is ubiquitously expressed in most human and mouse tissues. The TIPE mRNA and protein are mostly found in the bone marrow and immune system, gastrointestinal tract, lung, and adipose tissue in the human Protein Atlas database [13]. Accumulating evidences suggest that the expression of TIPE protein in cancers is generally higher when compared with adjacent normal tissues. Moreover, expression of TIPE is strongly associated with the development of various cancers including cancers of the liver, lung, breast, colon, prostate, and others by affecting cell proliferation, apoptosis, invasion, and metastasis [14–17]. However, its physiologic and pathologic functions in vivo are not clear until we generated *Tipe*^*−/−*^ mice [18, 19]. Although our previous work clearly indicated that loss of TIPE results in more severe acute DSS colitis [18], how TIPE is involved in the detailed mechanisms that regulate the inflammatory response is still unclear. Furthermore, no data are available on the role of TIPE in models of prolonged inflammation, such the colitis-induced carcinogenesis. In this study, we unveil an essential function of TIPE in restraining inflammation-associated gut microbiota dysbiosis and tumorigenesis in the CAC model.

## Materials and methods

### Mice

Wild type (WT) and *Tipe*^*−/−*^ mice on C57BL/6J (B6) background were generated by heterozygote crosses [18], and then the *Tipe*^*−/−*^ mice and their littermate mice were separately bred for experiments in different cages. CD45.1^+^ B6 mice were kindly provided by Dr. Yinming Liang (Xinxiang Medical University). Adult male 8–12-week-old mice were used except in antibiotic experiments where mice began antibiotic treatment at 5–6-week old. Cohousing experiments were also done with adult males. Group allocation for all experiments was randomized and not blinded. Sample analyses were not blinded. All mice used were maintained under pathogen-free conditions in the Xinxiang Medical University Animal Care Facilities. All animal procedures were preapproved by the Institutional Animal Care and Use Committee of the Xinxiang Medical University.

### Induction of colitis and CAC

Mice were injected intraperitoneally with 10 mg/kg azoxymethane (AOM, Sigma). Following AOM injection 5 days later, drinking water was supplemented with 2.0% or 2.5% (for survival studies) dextran sulfate sodium (DSS, m.w. 36–40 kDa; MP Biologicals) for 5 days, followed by regular drinking water for 16 days. Two additional cycles were used to repeat, and mice were sacrificed for phenotype analysis on day 90 of the experiments. For short-term colitis and inflammation studies, mice were also injected intraperitoneally with AOM. After 5 days, mice were administrated by 2% DSS for 5 days and euthanized at the indicated time points. Mice survival was monitored and body weight loss was recorded during DSS treatment and during regular water administration to measure disease progression. In addition, clinical Disease Activity Index (DAI) was determined as previously described [20], which consisted assessments of body weight loss, stool consistency, and fecal blood. At the time of euthanization, colons were removed from mice, flushed with cold PBS, opened longitudinally, and tumor burden was assessed by measuring the number and size of macroscopic tumors with a caliper.

### Bone marrow chimeras

Bone marrow chimeric mice were generated as previously described [20]. In brief, lethally irradiated (9.0 Gy) 7–8 week-old recipient mice were reconstituted by retro-orbital injection of 10 million bone marrow cells from age and gender matched donor mice. Recipient mice were administrated with antibiotic water for 2 weeks, followed by 6 weeks of regular water. Extent of reconstitution was assessed by flow cytometry for CD45.1/CD45.2 allele-expressing cells in the peripheral blood. Eight weeks after irradiation, chimeric mice were placed on the AOM/DSS protocol as described above.

### Antibiotic treatment and mice cohousing

For antibiotic treatment, mice were induced CAC with AOM/DSS. At 14 days after AOM administration, mice were placed on antibiotic mixture in autoclaved drinking water (ampicillin, 1 mg/ml; vancomycin, 0.5 mg/ml; neomycin, 1 mg/ml; and metronidazole, 1 mg/ml, all from Sangon Biotech). Fresh antibiotic was supplied every week. This antibiotic mixture was maintained for the duration of the experiment until mice were euthanized for tumor and tissue analysis.

For mice cohousing, age- and sex-matched WT and *Tipe*^*−/−*^ mice were weaned at the age of 4-week old from their parents, and were randomly separated into two cages. In control group, the mice were raised in separate cages to maintain their own microbiota composition, while in cohousing group, WT and *Tipe*^*−/−*^ mice were raised in the same cages at 1:1 ratio (one WT and one *Tipe*^*−/−*^ mouse in each cage). After 6 weeks, the mice were treated with AOM/DSS to induce acute colitis or CAC.

### Histological analysis and microscopy

Histological analysis was performed as previously described [20]. In brief, fresh colon pieces were fixed in 4% Paraformaldehyde solution and embedded in paraffin. Five-micron sections of paraffin-embedded colons were stained with hematoxylin and eosin (H&E) and analyzed for severity of inflammation and dysplasia. For immunohistochemical analysis, standard immunohistochemical procedures were performed using the following antibodies: β-catenin (Cell Signaling Technology; 8480) and Ki67 (Abcam, ab16667). Intratumoral apoptotic cells were detected in paraffin-embedded colon samples with the In Situ Cell Death Kit (Roche, 11684817910) according to the manufacturer’s instructions. After the immunohistochemical staining, slides were read under microscope and fields were randomly selected for further analysis. Images were taken and the number of positive cells in each field per high-power field within each tumor from WT and *Tipe*^*−/−*^ tumor-bearing mice were counted. At least three fields for each mouse were used for quantification, with the animal number of five for each group.

### Isolation of lamina propria cells and flow cytometry

Lamina propria (LP) cells of colon tissues were isolated as previously described [20]. Briefly, the fat, connective tissue and feces were first removed from colon, and then colon samples were added to Hanks’ Balanced Salt Solution (HBSS) buffer containing 30 mM EDTA/1 mM DTT at 37 °C with 220 RPM for 30 min for two cycles. Remaining colon pieces were washed with cold PBS and further digested for LP cell extraction in RPMI1640 medium (Corning) which contained collagenase VIII (Sigma, 0.5 mg/ml) and DNase I (Sigma, 100U/ml) at 37 °C with 120RPM for 90 min. After digestion, the supernatant containing LP cells was then passed through 70 μm mesh and cells were collected by centrifugation at 500 g for 5 min. Next, LP cells were incubated with purified anti-mouse CD16/CD32 antibody to block Fc receptor before surface staining. LP cells were then stained with monoclonal antibody mixes (all from Biolegend). Cells were acquired on the FACS Calibur or FACSCanto flow cytometer (BD Biosciences). Flow cytometry analysis was done with FlowJo software (v10.0; TreeStar).

### RNA isolation and real-time PCR

Total RNA of colon tissues was isolated using RNAiso Plus reagent (Takara) [21]. Five hundred nanograms of total RNA were reversely transcribed using RT Master Mix (Takara). Quantitative real-time PCR was performed in an Applied Biosystems 7500 System with TB Green Premix Ex Taq II (Takara). Gene transcript levels were normalized to actin. The primers used for real-time PCR assays were 5′-GCCTTCTTGGGACTGATGCT-3′ and 5′-CTGCAAGTGCATCATCGTTGT-3′ for *Il6*; 5′-CTCCAGAAGGCCCTCAGACTAC-3′ and 5′-AGCTTTCCCTCCGCATTGACACAG-3′ for *Il17a*; 5′-CCTCTAGCTGGAACACAGTGC-3′ and 5′-GCGGTTCTCATCTGTGTCG-3′ for *Il17c*; 5′-GACCAAACTCAGCAATCAGCTC-3′ and 5′-TACAGACGCAAGCATTTCTCAG-3′ for *Il22*; 5′-TCCCTACTAGGACTCAGCCAAC -3′ and 5′-TGGGCATCTGTTGGGTCT-3′ for *Il23p19*; 5′-AGTGTCCTCAGTTTGTGCAG-3′ and 5′-ACTCCTTGTGGCTGTCTTTG-3′ for *S100a8*; 5′-TTAAAAACCTGGATCGGAACCAA-3′ and 5′-GCATTAGCTTCAGATTTACGGGT-3′ for *Ccl2*; 5′-AGCAGTCCAACTCCGGGGAACAG-3′ and 5′-GTCGATCAGCGTGGTGGCGATG-3′ for *Tipe* and 5′-CCACACCCGCCACCAGTTCG-3′ and 5′-TACAGCCCGGGGAGCATCGT-3′ for *Actin*. The relative changes in gene expression were analyzed by the 2-Δct or 2-ΔΔct method, and specificity of qPCR amplification was assessed by melting curve analysis.

### Protein isolation and Western blotting

Colon tissue proteins were extracted with RIPA buffer (Beyotime) supplemented with protease and phosphatase inhibitors (Roche). Whole-tissue lysates were loaded and subjected to SDS-PAGE, transferred onto polyvinylidene difluoride (PVDF) membrane (Millipore), and then blotted by the Amersham Imager 600RGB detection system (GE Healthcare), as described previously [22]. Antibodies used are as follows: phospho-STAT3 (Cell Signaling Technology, 9145), phospho-P65 (Cell Signaling Technology, 3033), P65 (Cell Signaling Technology, 8242), phospho-Histone H2A.X (Cell Signaling Technology, 9718), Histone H2A.X (Cell Signaling Technology, 7631), STAT3 (Abclonal, A19566,), TIPE (Proteintech, 15790-1-AP,) and β-actin (Proteintech, 66009-1-Ig).

### 16S rRNA gene sequencing

16S ribosomal RNA (rRNA) analysis was performed with fecal samples that had been collected from mice and were frozen at −80 °C. Bacterial genomic DNA was isolated using a MagPure Soil DNA LQ Kit (Magen Bio Laboratories). The V4 region of the 16S rRNA gene was amplified using specific primers and sequenced on an Illumina Miseq PE300 system (OEBiotech Co., Ltd.). Paired-end sequences were merged to give an optimal alignment (overlap length ≥ 10 bp, mismatch proportion ≤ 20%). As an added quality control measure, the software package MacQIIME (version 1.9.1) pipeline was used to reduce sequencing errors and remove chimeras. Sequences were clustered into operational taxonomic units (OTUs) with a 97% similarity cutoff. Relative abundance of each OTUs and other taxonomic levels (from phylum to genus) was calculated for each sample to account for different levels of sampling across multiple individuals. Alpha diversity was calculated using the inverse Simpson index and the observed number of OTUs (richness). Principal coordinates analysis (PCoA) was performed by Emperor 0.9.4. Beta diversity analysis was used to evaluate differences of samples in commensal complexity. Beta diversity on both weighted and unweighted unifrac was calculated by QIIME software (Version 1.7.0). These sequencing data were deposited in the National Center for Biotechnology Information (NCBI) BioProject under accession number PRJNA816682. The SRA records will be accessible with the following link when they are released: https://www.ncbi.nlm.nih.gov/sra/ PRJNA816682.

### Bacterial DNA isolation and microbiota qPCR analysis

For isolation of mucosal bacterial DNA, the colon tissues were flushed vigorously and dissected longitudinally. The bacteria adherent to the mucosal surface and the inner mucus layer were collected in 1 ml sterile PBS. The mucosal bacteria were obtained by centrifugation at 5500 g for 10 min. Bacterial genomic DNA was extracted using Stool gDNA Miniprep Kit (Biomiga) according to the manufacturer’s instructions and subjected to qPCR with an Applied Biosystems 7500 Fast Real-time PCR system with TB Green Premix Ex Taq II (Takara). Relative abundances of specific taxa were normalized with the universal bacteria primer. The primers used for real-time PCR assays were 5′-ACTCCTACGGGAGGCAGCAGT-3′ and 5′-ATTACCGCGGCTGCTGGC-3′ for universal bacteria [23]; 5′-CATGACGTTACCCGCAGAAGAAG-3′ and 5′-CTCTACGAGACTCAAGCTTGC-3′ for Proteobacteria [24]; 5′-CMATGCCGCGTGTGTGAA-3′ and 5′-ACTCCCCAGGCGGTCDACTTA-3′ for Gammaproteobacteria [25]; 5′-GTGCCAGCMGCCGCGGTAA-3′ and 5′-GCCTCAAGGGCACAACCTCCAAG-3′ for Enterobacteriaceae [23]; 5′-CATGCCGCGTGTATGAAGAA-3′ and 5′-CGGGTAACGTCAATGAGCAAA-3′ for *E. coli* [23]; 5′-AGCAGTAGGGAATCTTCCA-3′ and 5′-CACCGCTACACATGGAG-3′ for *Lactobacillus* [23].

### Statistics

All quantitative data are presented as means ± SEM of two or three experiments. The survival curves were plotted according to the Kaplan–Meier method and compared by the log-rank test. A two-tailed Student’s *t* test was used for all other cases. The variance is similar between the groups. The *p* values < 0.05 were considered significantly. All statistical analysis was performed with the Prism 8.0 for Windows (Graphpad Software).

## Results

### Loss of TIPE promotes colitis-associated colorectal tumorigenesis

**Fig. 1:**
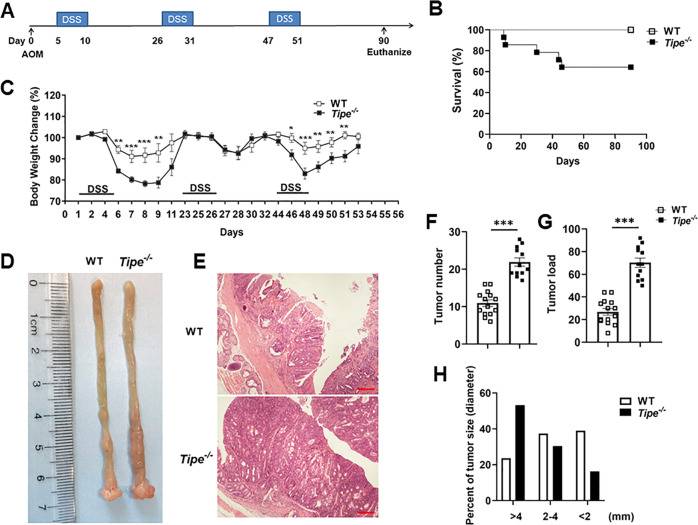
TIPE deficiency leads to increased tumorigenesis. **A** Schematic of AOM/DSS protocol for induction of colorectal tumor. **B** Survival curve of WT (*n* = 15) and *Tipe*^*−/−*^ (*n* = 15) mice undergoing AOM/DSS treatment. Data were pooled from three independent experiments. **C** Relative body weight change of WT (*n* = 5) and *Tipe*^*−/−*^ (*n* = 5) mice during AOM/DSS protocol. Data are representative of three independent experiments. **D**, **E** Representative macroscopic images (**D**) and H&E-stained sections (**E**) of colon tumors from WT (*n* = 5) and *Tipe*^*−/−*^ (*n* = 5) mice on day 90 of the CAC model. Scale bars, 100 μm; original magnification ×10. Data are representative of three independent experiments. **F**–**H** Colon tumor number (**F**), tumor load (**G**) and tumor-size distribution (**H**) of WT (*n* = 14) and *Tipe*^*−/−*^ (*n* = 12) mice on day 90 after AOM/DSS treatment. Data were pooled from three independent experiments. Data are presented as mean ± SEM. Kaplan–Meier analysis (**B**) (*p* < 0.05) or Student’s *t* test (**C**, **F**, and **G**), *p < 0.05, **p < 0.01, ***p < 0.001.

To investigate the role of TIPE in inflammation-associated colon tumorigenesis, WT and *Tipe*^*−/−*^ mice were subjected to a well-characterized CAC model (Fig. 1A). *Tipe*^*−/−*^ mice were extremely hypersensitive to AOM/DSS treatment, while *Tipe*^*−/−*^ mice demonstrated almost 40% mortality by the third DSS cycle (Fig. 1B). Due to the high mortality, we reduced the DSS dose to 2.0%. AOM/DSS-treated *Tipe*^*−/−*^ mice exhibited statistically greater body weight loss and delayed recovery compared to WT controls (Fig. 1C). After induction of tumorigenesis, mice were euthanized on day 90. There was no significant difference in the colon length between WT and *Tipe*^*−/−*^ mice (Supplemental Fig. 1A). However, spleens were significantly enlarged in tumor-bearing *Tipe*^*−/−*^ mice (Supplemental Fig. 1B, C). We also found that WT mice developed rectal prolapse, which was observed more often in *Tipe*^*−/−*^ mice (Supplemental Fig. 1D, E). As shown in Fig. 1D, macroscopic tumors occurred more frequently in *Tipe*^*−/−*^ mice than in WT mice. Histologically, in tumor-bearing *Tipe*^*−/−*^ mice, there were more low-grade adenocarcinomas with frequent invasion into submucosa and occasional invasion into muscularis propria (Fig. 1E). The overall tumor load was higher in *Tipe*^*−/−*^ mice compared to WT mice, mainly due to an increase in the number of tumors (Fig. 1F, G). Furthermore, analysis of tumor-size distribution showed that more than 50% of tumors in the colon of *Tipe*^*−/−*^ mice were larger in size (>4 mm diameter) (Fig. 1H). Thus, these observations suggest a potential tumor-suppressive function for TIPE in the development of CAC.

To further characterize TIPE-deficient colonic tumors, we then conducted immunohistochemical analyses. We observed uniform intense staining in *Tipe*^*−/−*^ tumors, whereas WT tumors often exhibited regional or subregional variation in β-catenin staining intensity (Supplemental Fig. 2A). Quantification with a previously reported β-catenin staining index demonstrated higher scores in *Tipe*^*−/−*^ tumors compared with WT tumors (Supplemental Fig. 2A). We also found proliferation was increased in *Tipe*^*−/−*^ tumors as measured by Ki67 staining (Supplemental Fig. 2B). However, intratumoral apoptosis determined by TUNEL did not show significant differences between tumors from WT and *Tipe*^*−/−*^ mice (Supplemental Fig. 2C). Based on these data, the cellular homeostasis is altered in *Tipe*^*−/−*^ tumors, and the balance among proliferation, apoptosis, and β-catenin activity, a known driver of CRC pathogenesis is skewed towards tumor growth.

### TIPE controls the tumor-promoting inflammatory microenvironment

**Fig. 2:**
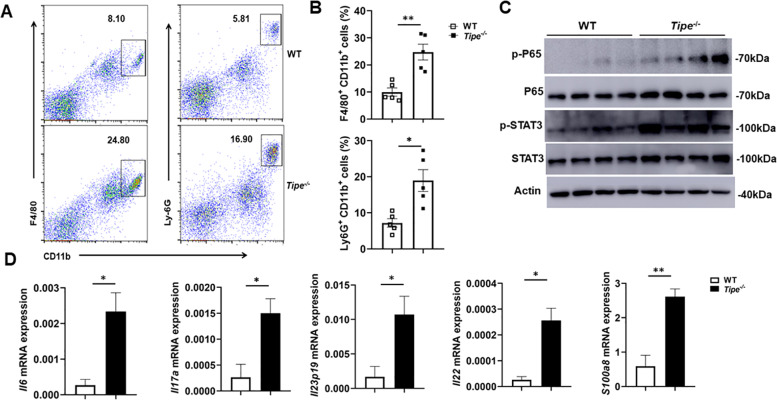
The inflammation is increased within colon tumors of *Tipe*^*−/−*^ mice. **A** Flow cytometic dot plot analysis of CD45^+^F4/80^+^CD11b^+^ cells and CD45^+^Ly-6G^+^CD11b^+^ cells in colon tumor tissues from WT (*n* = 5) and *Tipe*^*−/−*^ (*n* = 5) mice on day 90 of the CAC model. **B** Quantification of CD45^+^F4/80^+^CD11b^+^ cells and CD45^+^Ly-6G^+^CD11b^+^ cells in colon tumor tissues as described in (**A**). **C** Western blot analysis of p-P65 and p-STAT3 in the colon tumor tissues of WT (*n* = 4) and *Tipe*^*−/−*^ (*n* = 4) mice on day 90 of the CAC model. Blots represent data from the same biological samples run in parallel. **D** Quantification of inflammatory genes *Il6*, *Il17a*, *Il22*, *Il23p19* and *S100a8* mRNA expression by quantitative real-time PCR in the colon tumor tissues of WT (*n* = 5) and *Tipe*^*−/−*^ (*n* = 5) mice on day 90 of the CAC model. Data are presented as mean ± SEM and are representative of three independent experiments. Student’s *t* test (**B**, **D**), *p < 0.05, **p < 0.01.

Inflammation can function as a critical driver of tumorigenesis. Therefore, we first examined the immune cells infiltration. Tumors in *Tipe*^*−/−*^ mice showed more macrophages and neutrophils infiltration compared to those in WT mice (Fig. 2A, B). We also evaluated the activation status of transcription factors NF-κB and STAT3, potent activators of inflammatory pathways that contribute to oncogenic signaling leading to enhanced cell proliferation and tumor growth [26]. We found that activation of both transcription factors, as well as the expression of proinflammatory genes, was much increased in the tumors of *Tipe*^*−/−*^ mice (Fig. 2C, D). Thus, these results show that TIPE deficiency leads to an inflammatory milieu marked by enhanced pro-tumorigenic signaling pathways.

Since both AOM and DSS contribute to the development of CAC, we next performed DSS alone and AOM alone experiments. In agreement with our previous report [18], we found that *Tipe*^*−/−*^ mice were more susceptible to DSS-induced colitis, as indicated by greater body weight loss, a higher disease activity index and shorter colon lengths than WT mice upon DSS treatment (Supplemental Fig. 3A–C). In line with the increased severity of colitis indicators, the large areas of complete crypt loss and erosions were observed in colons from DSS-treated *Tipe*^*−/−*^ mice (Supplemental Fig. 3D). In chronic colitis mouse model, WT and *Tipe*^*−/−*^ mice were treated with three cycles of DSS without AOM and no tumor formation was observed (Supplemental Fig. 3E), indicating that DSS-induced colitis was not sufficient to induce tumor development. Furthermore, we also monitored the tumor formation induced by AOM without DSS for six months and found a single AOM injection did not develop tumors (Supplemental Fig. 3F). Taken together, loss of TIPE promotes tumor formation in an inflammatory carcinogenesis challenge.

### TIPE restrains inflammation-associated dysbiosis during early stages of colorectal tumorigenesis

Microbiota-induced inflammation is critical for the regulation of intestinal homeostasis, which promotes us to analyze the intestinal microbiota of mice treated with AOM/DSS at day 0 (steady state) and at day 14 (early stages of CAC) by 16 S rRNA gene profiling of fecal specimens. The relative abundance of bacteria at the phylum level and the species composition cluster according to Operational Taxonomic Units (OTUs) between naïve WT and *Tipe*^*−/−*^ mice was similar (Supplemental Fig. 4A). Furthermore, at day 0 there was no difference in α diversity (Supplemental Fig. 4B). These data suggest that increased susceptibility to DSS-induced colitis and CRC development in *Tipe*^*−/−*^ mice is independent of TIPE deficiency-caused gut microbiota shifts.

**Fig. 3:**
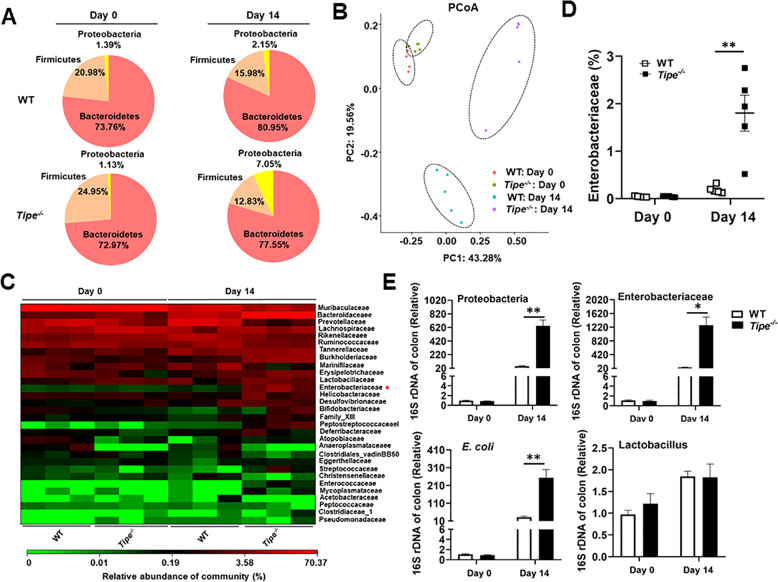
TIPE deficiency accelerates inflammation-associated dysbiosis. **A** Comparison of fecal specimens bacterial communities from WT (*n* = 5) and *Tipe*^*−/−*^ (*n* = 5) mice on day 0 or day 14 of the CAC model at the top 3 phylum level by 16S rRNA sequencing. **B** PCoA analysis of microbiome in fecal specimens from WT (*n* = 5) and *Tipe*^*−/−*^ (*n* = 5) mice on day 0 or day 14 of the CAC model by 16S rRNA sequencing. **C** Heatmap depicting of relative abundance of family level microbiota in the feces as in (**B**) (*n* = 3 per group). **D** Quantification analysis of Enterobacteriaceae family microbiota distribution in the feces as in (**B**) by 16S rRNA sequencing (*n* = 5 per group). **E** Quantitative PCR analysis of 16S rRNA genes of Proteobacteria phylum, Enterobacteriaceae family, *E. coli* and *Lactobacillus* species at colon mucosal surface of WT and *Tipe*^*−/−*^ mice on day 0 or day 14 of the CAC model (*n* = 3 or 5 per group). Data are presented as mean ± SEM and are representative of three independent experiments. Student’s *t* test (**D**, **E**), *p < 0.05, **p < 0.01.

Intestinal inflammation is accompanied by disruption of the gut microbiota (dysbiosis). A common feature of bacterial dysbiosis is an expansion of the population of Proteobacteria, in particular members of the Enterobacteriaceae family [27]. We hypothesized that TIPE controls inflammation-associated dysbiosis during early stages of AOM/DSS treatment, which ultimately determines host susceptibility to colorectal tumorigenesis. After the treatment (day 14), *Tipe*^*−/−*^ mice displayed lower OUT numbers and Shannon index compared with WT mice (Supplemental Fig. 5A, B). This may be explained by the excessive immune response generated by higher inflammation, which resulted in reducing bacterial richness. Bacterial communities differed significantly between samples of WT and *Tipe*^*−/−*^ mice (Fig. 3A, B). Further analyses showed that the abundance of Proteobacteria phylum bacteria was the most dramatically increased among these six phyla in *Tipe*^*−/−*^ mice (Fig. 3, Supplemental Fig. 5C, D). When viewed at the family level, the most notable feature between WT and *Tipe*^*−/−*^ mice was the dramatic difference in Enterobacteriaceae after the induction, a large family of gram-negative facultative bacteria within the phylum Proteobacteria (Fig. 3C, D and Supplemental Fig. 5E). We then confirmed the upregulation of Proteobacteria, Enterobacteriaceae and *E. coli*, the genera of Enterobacteriaceae, by qPCR in the colon mucosal surface of both WT and *Tipe*^*−/−*^ mice (Fig. 3E). In contrast, no significant differences were found in *Lactobacillus* between these groups (Fig. 3E). Thus, our data suggest that deficiency of TIPE results in more severe dysbiosis and consequently further worsened colitis pathology during early stages of colorectal tumorigenesis.

### Tumor-bearing *Tipe*^*−/−*^ mice exhibit altered microbial landscape

**Fig. 4:**
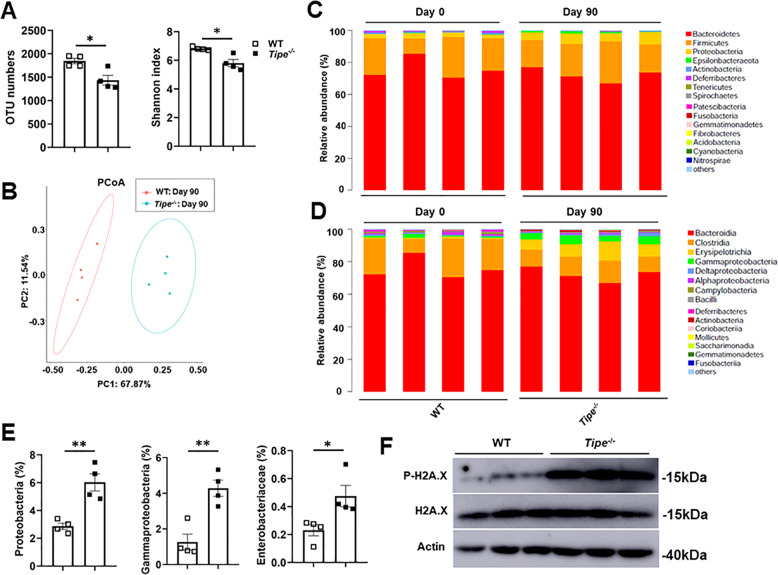
The intestinal microbiota is altered in tumor-bearing *Tipe*^*−/−*^ mice. **A** Operational taxonomic unit (OTU) abundances (left) and Shannon-Wiener diversity index (Shannon index) (right) of the feces from WT (*n* = 4) and *Tipe*^*−/−*^ (*n* = 4) mice on day 90 of the CAC model by 16S rRNA sequencing. **B** PCoA analysis of microbiome in the feces from WT (*n* = 4) and *Tipe*^*−/−*^ (*n* = 4) mice on day 90 of the CAC model by 16 S rRNA sequencing. **C**, **D** Relative abundance of microbiota at the top 15 phylum level (**C**), or the top 15 class level (**D**) in the feces by taxon-based analysis as in (**A**) (*n* = 4 per group). **E** Quantification analyses of Proteobacteria phylum, Gammaproteobacteria class and Enterobacteriaceae family microbiota distribution in the feces as in (**A**) (*n* = 4 per group). **F** Western blot analysis of p-H2A.X in WT (*n* = 3) and *Tipe*^*−/−*^ (*n* = 3) colon tumors on day 90 of the CAC model. Blots represent data from the same biological samples run in parallel. Data are presented as mean ± SEM and are representative of three independent experiments. Student’s *t* test (**A** and **E**), *p < 0.05, **p < 0.01.

Next, we explored the diversity and composition of microbiota after induction of tumorigenesis. The 16S rRNA gene sequencing in feces showed that there were significant differences in α-diversity between WT and *Tipe*^*−/−*^ mice (Fig. 4A). Moreover, we found that samples of *Tipe*^*−/-*^ mice clustered separately from WT in bray curtis plot (Fig. 4B), indicating that significant changes in the fecal microbiota composition. The relative abundance of specific bacterial taxa was analyzed at the level of phylum, class, and family. The abundance of the phylum Proteobacteria and the class Gamma-proteobacteria exhibited significant increase in tumor-bearing *Tipe*^*−/−*^ mice (Fig. 4C–E). Similar difference was also observed for the family Enterobacteriaceae (Fig. 4E). Enterobacteriaceae evidenced a significant increase of this family bacterium in *Tipe*^*−/−*^ mice, while it was almost undetectable at day 0. This is consistent with previous report that Enterobacteriaceae are among the most commonly overgrown symbionts in colorectal cancer as a result of inflammation in the gut conferring a growth advantage to Enterobacteriaceae [28]. Tumor-associated bacteria can induce intratumoral genetic instability by promoting DNA double-stranded breaks [29]. *Tipe*^*−/−*^*-*derived tumors had significantly more phosphorylated histone p-H2A.X, indicative of enhanced intratumoral genetic instability (Fig. 4F). Taken together, these data show that TIPE controls the gut microbial landscape and that the deficiency of TIPE appears to provide a favorable environment for expansion of Enterobacteriaceae.

### Colon microbiota are essential for increased tumor growth in *Tipe*^*−/−*^ mice

The microbiota have been strongly associated with the initiation and progression of CAC [30]. To evaluate whether the microbiota could have an impact on the colitis and CAC development, we performed cohousing experiments. WT and *Tipe*^*−/−*^ mice were cohoused at a 1:1 ratio for 6 weeks before and throughout these experiments (Supplemental Fig. 6A). It was evident that the cohousing strategy had an obvious effect: both cohoused WT and *Tipe*^*−/−*^ mice displayed reduced body weight loss (Supplemental Fig. 6B); furthermore, colon length did not differ between cohoused WT and *Tipe*^*−/−*^ mice (Supplemental Fig. 6C). Similar results were also observed in tumor number and tumor load (Supplemental Fig. 6D, E), suggesting that endogenous microbiota play a critical role in the increased tumor growth in mono-housed *Tipe*^*−/−*^ mice.

**Fig. 5:**
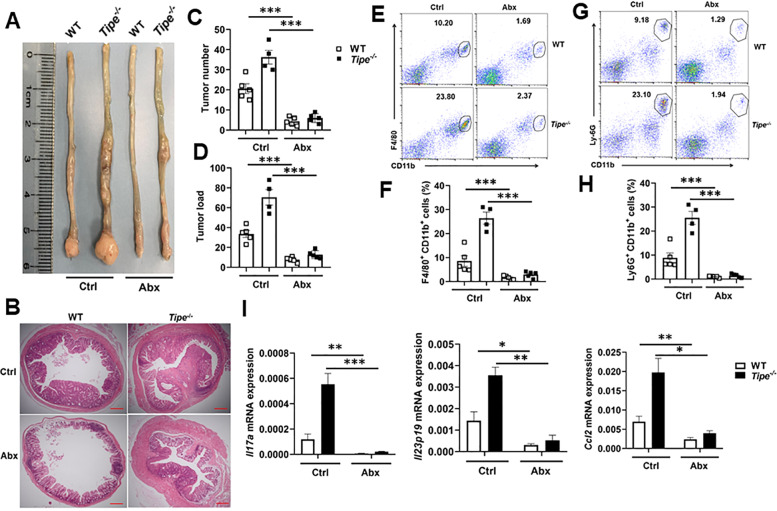
The microbiota promotes tumor progression in *Tipe*^*−/−*^ mice. **A**, **B** Representative macroscopic images (**A**) and H&E-stained sections (**B**) of colonic tissue with tumors from WT and *Tipe*^*−/−*^ mice treated with antibiotics (Abx) or no antibiotics (Ctrl) on day 90 of the CAC model (*n* = 4 or 5 per group). Scale bars, 100 μm; original magnification ×4. **C**, **D** Colon tumor number (**C**) and tumor load (**D**) from WT and *Tipe*^*−/−*^ mice as described in (**A**). **E**, **F** Flow cytometic dot plot analysis (**E**) and quantification of CD45^+^F4/80^+^CD11b^+^ cells (**F**) in colon tumor tissues from WT and *Tipe*^*−/−*^ as described in (**A**). **G**, **H** Flow cytometic dot plot analysis (**G**) and quantification of CD45^+^Ly-6G^+^CD11b^+^ cells (**H**) in colon tumor tissues from WT and *Tipe*^*−/−*^ mice as described in (**A**). **I** Quantification of inflammatory genes *Il17a*, *Il22,* and *ccl2* mRNA expression by quantitative real-time PCR in the colon tumor tissues of WT and *Tipe*^*−/−*^ mice treated with Abx or Ctrl on day 90 of the CAC model (*n* = 4 or 5 per group). Data are presented as mean ± SEM and are representative of two independent experiments. Student’s *t* test (**C**, **D**, **F**, **H**, **I**), *p < 0.05, **p < 0.01, ***p < 0.001.

Next, we applied the antibiotic-treatment mouse model. Although *Tipe*^*−/−*^ mice developed dramatically larger colon tumors than WT mice, antibiotic treatment alleviated tumor formation (Fig. 5A, B), and decreased the tumor number and tumor load in *Tipe*^*−/−*^ mice to the levels of those of WT mice (Fig. 5C, D). Flow cytometric analyses exhibited robust infiltration of tumor-associated macrophages and neutrophils into primary tumor tissues. Likewise, antibiotic treatment decreased tumor-associated macrophages and neutrophils in *Tipe*^*−/−*^ mice to the levels of those of WT mice (Fig. 5E–H). Similar reduction was also noted for inflammatory genes *Il17a*, *Il22* and *ccl2* (Fig. 5I). These data define an important contribution of microbiota in enhancing tumor growth and progression observed in *Tipe*^*−/−*^ mice.

To further confirm the role of microbiota in tumor development, WT recipient mice were gavaged with feces from tumor-bearing WT or *Tipe*^*−/−*^ mice. We found the mice that received microbiota from tumor-bearing *Tipe*^*−/−*^ mice developed significantly more tumors, compared with mice received microbiota from tumor-bearing control mice (Supplemental Fig. 6F, G). These data suggest that the altered microbiota in *Tipe*^*−/−*^ mice is responsible for the increased CAC development.

### TIPE deficiency in hematopoietic cells does not promote tumorigenesis

**Fig. 6:**
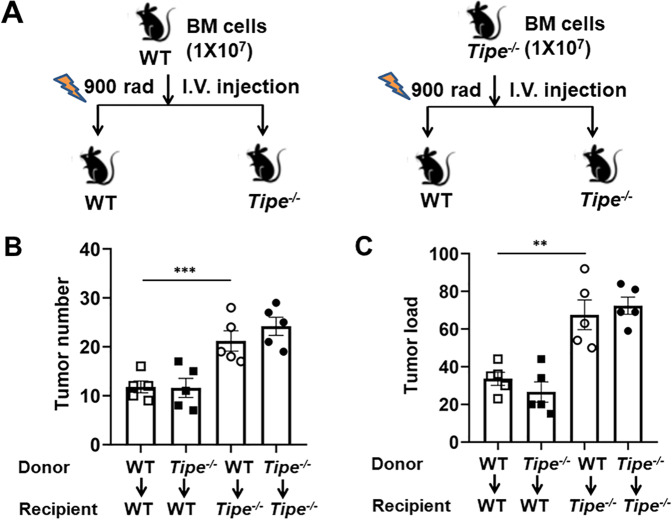
Restoration of hematopoietic TIPE fails to rescue *Tipe*^*−/−*^ AOM/DSS phenotype. Bone marrow transplants were performed on WT and *Tipe*^*−/−*^ mice as indicated. **A** Schematic of experimental design. WT and *Tipe*^*−/−*^ mice were irradiated, transferred with WT or *Tipe*^*−/−*^ bone marrow cells, allowed to recover 8 weeks, and followed by AOM/DSS protocol. **B**, **C** Colon tumor number (**B**) and tumor load (**C**) of BM-transplanted WT and *Tipe*^*−/−*^ as described in (**A**) (*n* = 5 per group). Data are representative of two independent experiments. Data are presented as mean ± SEM and are representative of two independent experiments. Student’s *t* test (**B**, **C**), **p < 0.01, ***p < 0.001.

TIPE is ubiquitously expressed in various tissues and is an important regulator of the intestinal injury response [19]. To evaluate whether the increased tumorigenesis observed in *Tipe*^*−/−*^ mice was due to a TIPE hematopoietic cell-autonomous effect, we performed bone marrow (BM) transplantation experiments: *Tipe*^*−/−*^ mice transplanted with BM from WT mice (WT > *Tipe*^*−/−*^), WT mice transplanted with BM from *Tipe*^*−/−*^ mice (*Tipe*^*−/−*^ > WT), and the two control groups consisting of WT mice transplanted with BM from WT mice (WT > WT), and *Tipe*^*−/−*^ mice transplanted with BM from *Tipe*^*−/−*^ mice (*Tipe*^*−/−*^ > *Tipe*^*−/−*^) (Fig. 6A). The transfer of WT BM into *Tipe*^*−/−*^ recipients failed to rescue the *Tipe*^*−/−*^ AOM/DSS phenotype of increased tumorigenesis. *Tipe*^*−/−*^ mice receiving WT BM and WT mice receiving *Tipe*^*−/−*^ BM phenocopied the *Tipe*^*−/−*^ mice receiving *Tipe*^*−/−*^ BM and WT mice receiving WT BM, respectively, in terms of tumor number and tumor load (Fig. 6B, C). Additionally, *Tipe*^*−/−*^ mice receiving *Tipe*^*−/−*^ BM exhibited increased tumor number and tumor load when compared with WT mice receiving WT BM (Fig. 6B, C). These results demonstrate that TIPE deficiency in hematopoietic lineages is likely not responsible for the pro-tumorigenic phenotype in *Tipe*^*−/−*^ mice.

### TIPE expression is reduced in human colorectal cancer

**Fig. 7:**
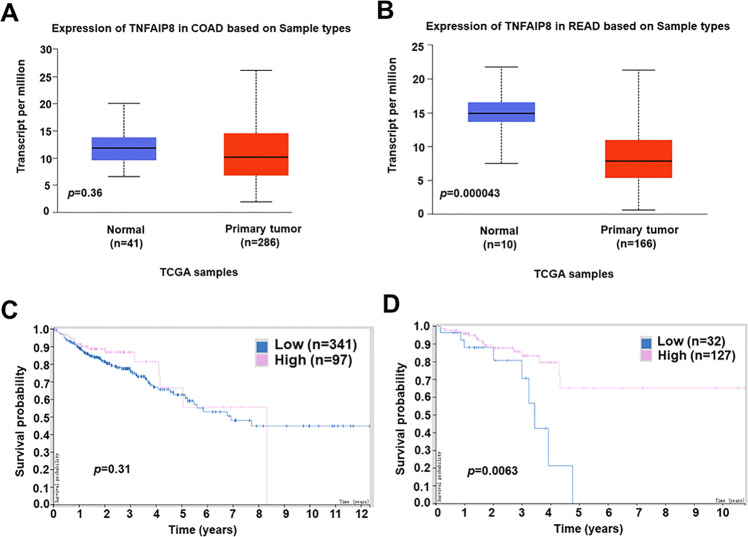
*TIPE* gene expression is downregulated in human colorectal cancer and association of *TIPE* expression with patient survival. **A** Expression of *TIPE* in colon adenocarcinoma (COAD) compared to matched TCGA normal tissue. **B** Expression of *TIPE* in rectum adenocarcinoma (READ) compared to matched TCGA normal tissue. **C**, **D** Association of *TIPE* expression with patient survival in colon adenocarcinoma (**C**) or rectum adenocarcinoma (**D**) revealed through analyses of the human Protein Atlas database.

To determine whether TIPE was dysregulated in human CRC, we examined *TIPE* mRNA levels in colon adenocarcinoma (COAD) and in rectum adenocarcinoma (READ) through analyses of TCGA database using the UALCAN web server. Intriguingly, the downregulation of *TIPE* was not observed in COAD (Fig. 7A). However, analysis of READ showed that the expression of *TIPE* mRNA was significantly reduced as compared to normal adjacent tissue (Fig. 7B). When we analyzed the human Protein Atlas database, we found the association of *TIPE* low expression with worsened patient survival in READ, but not in COAD (Fig. 7C, D). We next further evaluated the translational relevance of our findings by analyzing *TIPE* expression with the Tumor Immune Single- cell Hub (TISCH). There are eight CRC datasets in total at this time, including five human CRC datasets (Supplemental Fig. 7A). We found that only CRC_GSE146771_Smartseq2 applied FACS to isolate CD45^+^ (immune fractions) and CD45^-^ (non-immune fractions), so this dataset was chosen to analyze *TIPE* gene expression. Among these cell populations, immune cells showed the highest expression of the *TIPE* gene compared with malignant and stromal cells (Supplemental Fig. 7B, C). Major-lineage cell clusters identified from this dataset and *TIPE* gene distribution pattern could be visualized by a combined t-distributed stochastic neighbor embedding (t-SNE) analysis (Supplemental Fig. 7D). Collectively, these data show that TIPE is reduced in human colorectal cancer and may contribute to its pathogenesis.

## Discussion

To date, we have learned some biological functions of TIPE through the analyses of phenotype of *Tipe*^*−/−*^ mice. In addition to regulating *Listeria monocytogenes* infection [31], these mice exhibited more sever colitis after DSS-induced injury [18], increased enterocyte proliferation, altered intestinal differentiation, protection against ischemia-induced and radiation-induced intestinal injury [19], and exacerbated gut acute graft-versus-host disease [32], indicating that TIPE is a key contributor to intestinal homeostasis. Based on the exacerbation of acute colitis and alteration in epithelial restitution identified in the setting of loss of TIPE, we hypothesized that TIPE may play a crucial role in modulating the risk for colorectal tumorigenesis as the inflammatory response is thought to drive the development of tumors. Here, we show that TIPE suppresses the formation of colorectal tumors in a model of inflammation-associated carcinogenesis. Our study identifies TIPE as a bona fide tumor suppressor in mice.

There are now ample evidences for a critical role of chronic inflammation in the etiology of many cancers. This association is particularly notable in CRC, as CRC occurs at the largest interface between host and intestinal microbial communities and is often associated with an inflammatory response, which is very different from many other types of cancer that develop in a relatively sterile environment [33]. Chronic inflammation promotes cancer progression by increasing the production of pro-tumorigenic cytokines, mainly IL-6, IL-17A, IL-22 and IL-23, which activate NF-κB and STAT3 signaling pathways [34]. In the AOM/DSS-induced CAC model, we found that inflammation was increased in *Tipe*^*−/−*^ mice at an early stage of tumor development, which could be a result of deficits in epithelial regeneration after DSS injury as previously reported [19]. This is in line with other study showing an inhibitory effect of TIPE on inflammation [32]. Consistent with inflammatory condition of early stage, enhanced inflammation was also observed in the *Tipe*^*−/−*^ tumor tissues. It is possible that the enhanced inflammation late in the *Tipe*^*−/−*^ tumor is due to enhanced “tumor-elicited inflammation.” These data indicate that inflammation may be the initial cause leading TIPE to regulate intestinal tumorigenesis. Because of the relative higher expression of TIPE in the bone marrow and immune system, we had assumed promotion of a pro-tumorigenic microenvironment was due to loss of TIPE in immune cells. However, this phenotype is not an immune cell autonomous. This implies that epithelium-derived TIPE might influence the recruitment or differentiation of immune cells in tumorigenesis. TIPE2, another well-studied member of the TNFAIP8 family, is restricted to the immune compartment. TIPE and TIPE2 have functional overlap in immune cells, and differences between the TIPE and TIPE2 knockouts help us elucidate the immune effects of loss of TIPE2 and the non-immune effects of loss of TIPE. It is possible that TIPE plays redundant roles in controlling immune cells function in the presence of TIPE2 during the development of CAC. One way to approach this question and to identify key molecular pathways linking specific arms of inflammation to tumorigenesis is to perform analysis of mice with tissue-specific gene deletion. We are in the process of generating a condition *Tipe* allele to formally test the epithelial- and immune cell-derived contributions of TIPE in inflammation and CAC.

Gut microbiota have been related to multiple diseases, including colitis and colon cancers [35, 36]. CRC-associated microbiota were found to contribute to oncogenic epigenetic alterations in colonic mucosa [37]. In our study, we analyzed the bacterial taxonomic composition of intestinal microbiota of WT and *Tipe*^*−/−*^ mice at steady state and found no differences. However, at the end of experiment, *Tipe*^*−/−*^ mice exhibited different bacterial composition with reduced diversity. The 16S rRNA sequencing analysis unveiled that a significant difference was found for Proteobacteria/Enterobacteriaceae. Blooms of Enterobacteriaceae have been reported to associate with colitis and CRC and contribute to its pathogenesis [28]. It is in agreement with the results of our study showing higher abundance of Enterobacteriaceae, which means higher tumor induction in *Tipe*^*−/−*^ mice. To investigate the role of microbiota in tumorigenesis, we did cohousing experiments. The results clearly indicated that the mixed composition of WT and *Tipe*^*−/−*^ mice microbiome had a positive effect in limiting tumor development, and tumor number and tumor load were ameliorated in *Tipe*^*−/−*^ mice. The difference in tumor number and tumor load also vanished. The crucial role of microbiota in tumorigenesis was further confirmed by antibiotic-treatment assay and gut microbiota transferring experiment. Therefore, the dysregulation observed in *Tipe*^*−/−*^ mice may be the consequence of their inability to repair the intestinal barrier due to defective epithelial regeneration upon injury [19] and to appropriately regulate the commensal microbiota expansion and intestinal inflammation.

In summary, we extend our understanding of TIPE beyond its known roles in tumorigensis by identifying it as a tumor suppressor in inflammatory carcinogenesis. To the best of our knowledge, this is the first study which explores its role in tumors arising from endogenous tissue. TIPE regulates epithelial proliferation and regeneration, inflammation-associated gut microbiota dysbiosis, and the tumor inflammatory microenvironment, all of which affect tumor growth and progression. Moreover, TIPE appears to contribute to human disease, as TIPE was reduced in human colorectal cancer patients. Our results also suggest that TIPE may be a tumor suppressor in a diverse spectrum of epithelium-based malignancies.

## Supplementary information

Supplementary Information

Reproducibility checklist

Uncropped original western blots

## Data Availability

All data needed to evaluate the conclusions of this paper are already present in the article file and supplemental information. Additional related data need to be requested from the corresponding author.
