# Supplementary material for: TNFAIP8 protein functions as a tumor suppressor in inflammation-associated colorectal tumorigenesis
Source: Cell Death Dis. 2022 Apr 6;13(4):311. doi: 10.1038/s41419-022-04769-x (PMC8986800; doi:10.1038/s41419-022-04769-x)
Supplement: Supplementary file 1 — Supplementary Information [file 41419_2022_4769_MOESM1_ESM.docx]

**Supplementary Information**


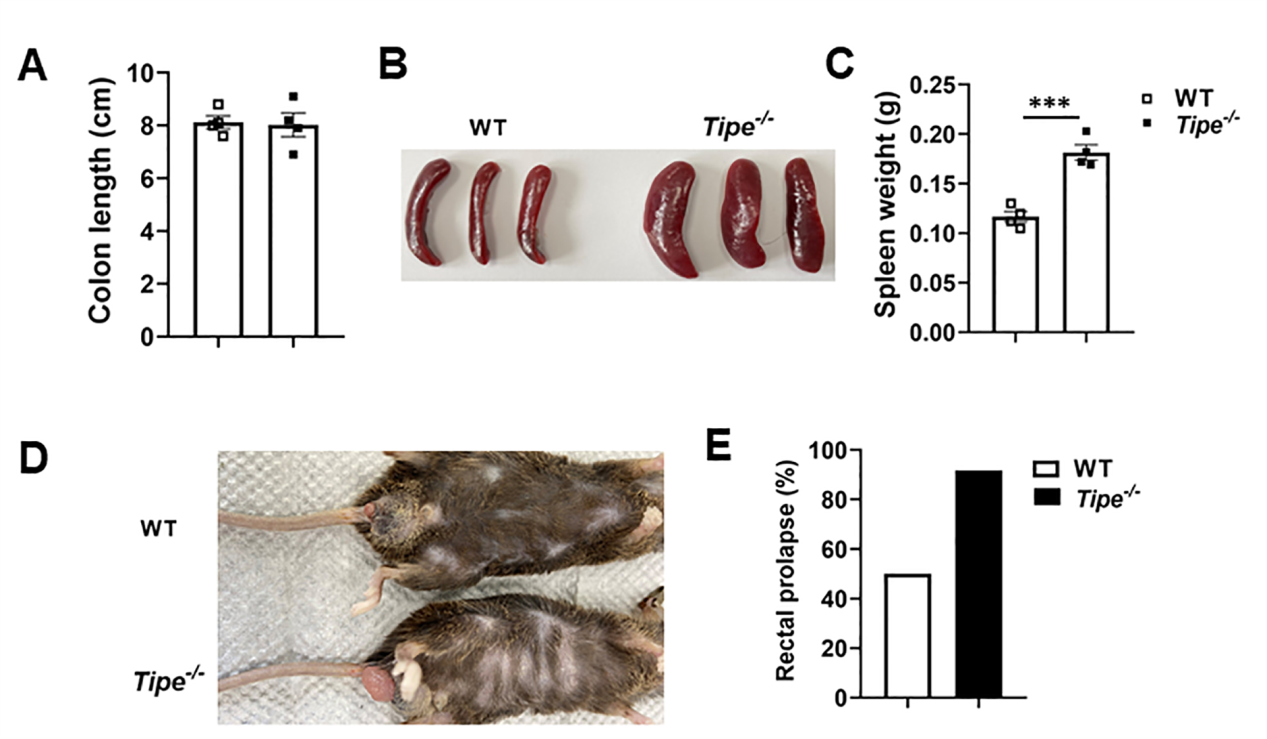


**Supplemental Figure 1.** Phenotype analyses of the CAC model in WT and *Tipe^-/-^* mice. **A** Colon length of WT (n=4) and *Tipe^-/-^* (n=4) mice on day 90 of the CAC model. Data are representative of three independent experiments. **B-C** Representative images of spleen (**B**) and spleen weight (**C**) as described in **A**. Data are representative of three independent experiments. **D-E** Representative images showing rectal prolapse (**D**) and percentage of rectal prolapse (**E**) observed in WT (n=14) and *Tipe^-/-^* (n=12) mice. Data were pooled from three independent experiments. Data are presented as mean± SEM. Student’s t test (**A** and **C**), ****p*<0.001.


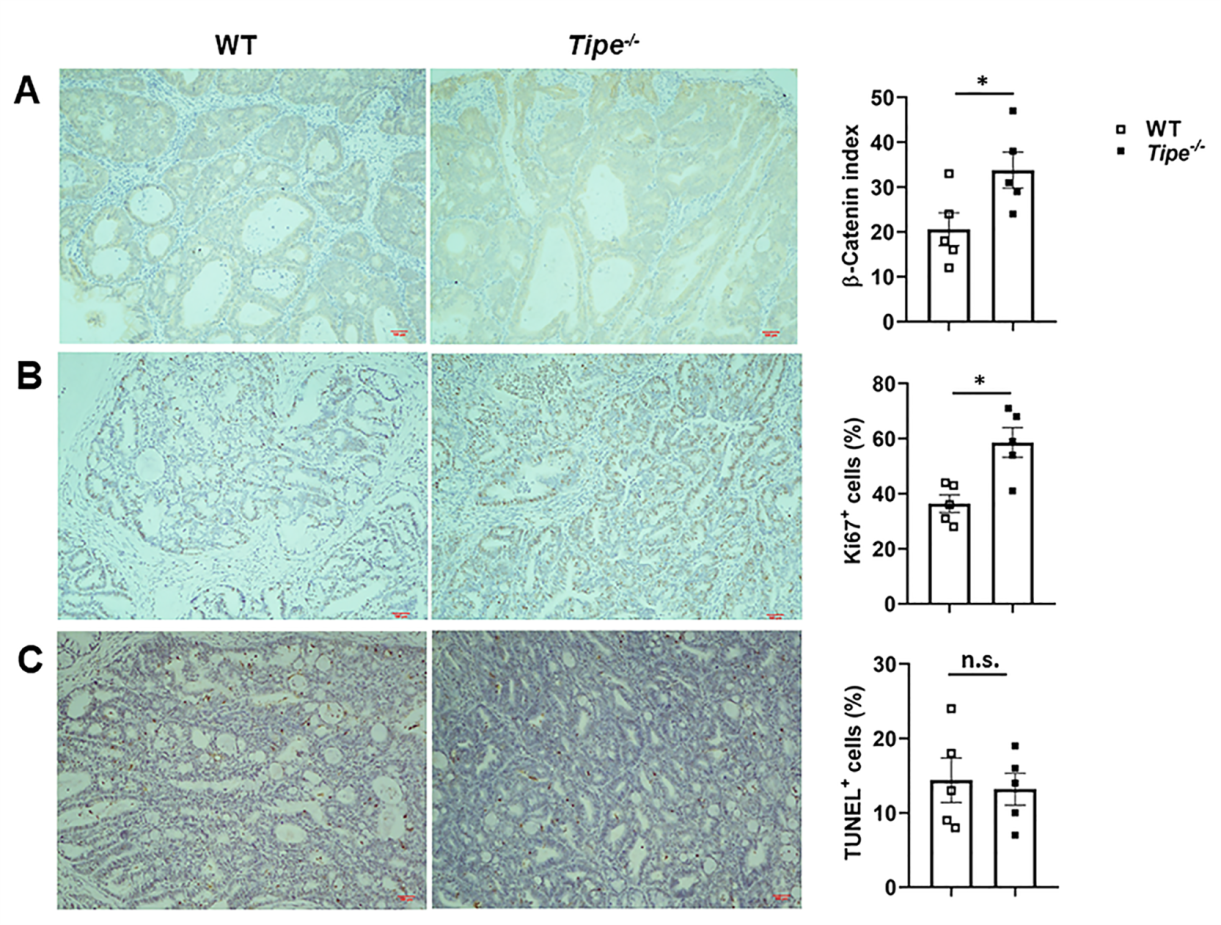


**Supplemental** **Figure 2.** The expression and heterogeneous distribution of β-catenin and proliferation are increased in *Tipe^-/-^* tumors. **A** Representative images from WT and *Tipe^-/-^* tumors stained for β-catenin (left) and quantification of β-catenin staining index (right, percentage of tumors with β-catenin multiplied by expression intensity) (n=5 per group). Scale bars, 100 μm; original magnification × 10. **B** Representative images from Ki67-labeled tumors (left) and quantification of intratumoral proliferation (right, percentage of Ki67^+^ cells) (n=5 per group). Scale bars, 100 μm; original magnification × 10. **C** Representative images from TUNEL-labeled tumors (left) and quantification of intratumoral apoptosis (right, percentage of TUNEL^+^ cells) (n=5 per group). Scale bars, 100 μm; original magnification × 10. Data are presented as mean± SEM and are representative of three independent experiments. Student’s t test (right, **A**, **B** and **C**), **p*<0.05.


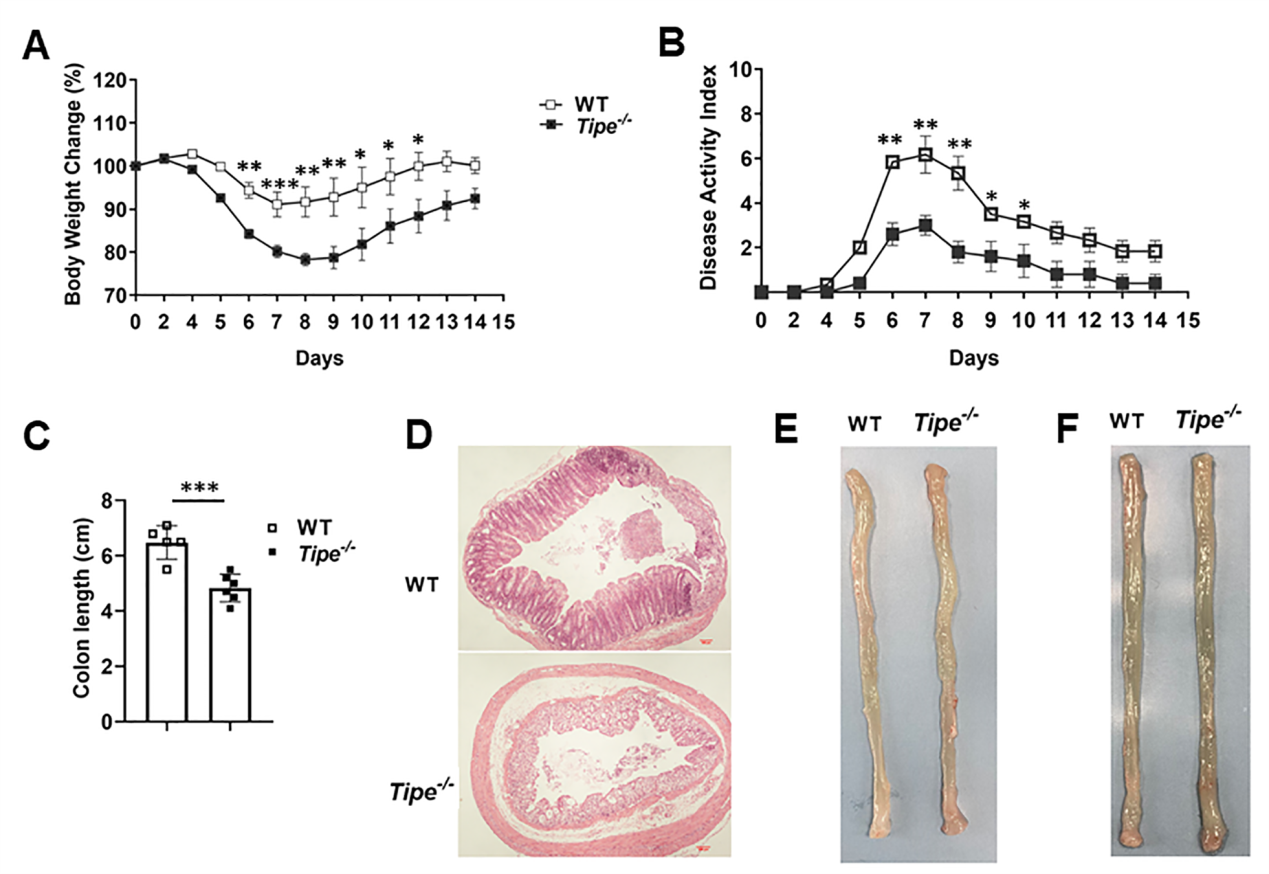


**Supplemental Figure 3.** *Tipe^-/-^* mice are more susceptible to DSS-induced colitis and epithelial damage. **A-B** Body weight changes (**A**), and disease activity index (**B**) in WT (n=5) and *Tipe^-/-^* (n=6) mice during administration of 2.0% DSS in drinking water after AOM injection. **C** Measurement of colon length of WT and *Tipe^-/-^* mice on day 14 of the CAC model as described in **A**. **D** Representative H&E-stained colon sections from mice as described in **C**. Scale bars, 100 μm; original magnification × 4. **E** WT (n=4) and *Tipe^-/-^* (n=5) mice were given AOM on day 1 and were kept till six months without DSS administration. Mice were then euthanized on day 180. Representative macroscopic images of colon were shown. **F** WT (n=5) and *Tipe^-/-^* (n=5) mice were given three cycles of 2% DSS and were kept till six months without AOM administration. Representative macroscopic images of colon were shown. Data are presented as mean± SEM and are representative of three independent experiments. Student’s t test (**A**, **B** and **C**), **p*<0.05, ***p*<0.01, ****p*<0.001.


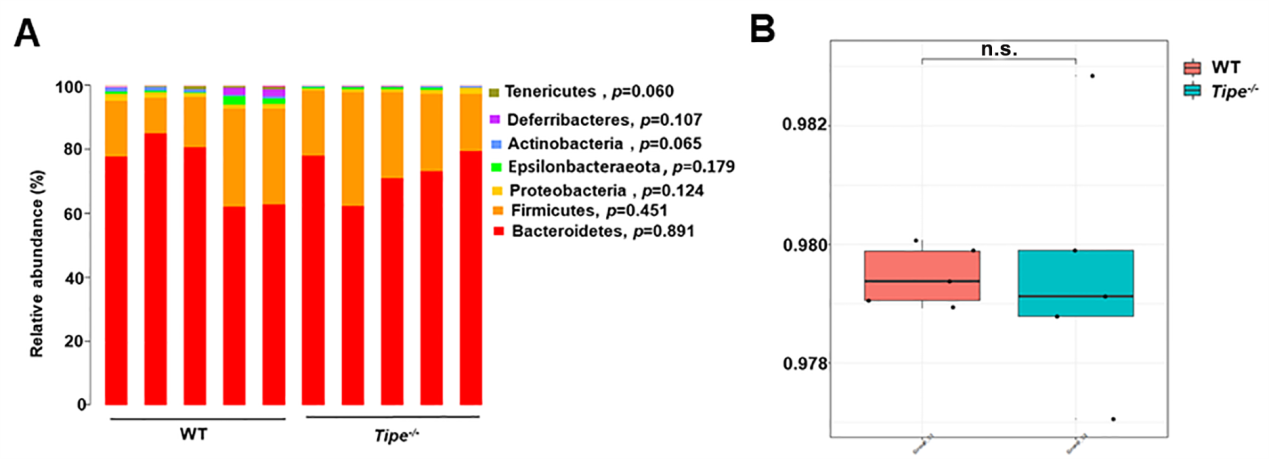


**Supplemental** **Figure 4**. Microbiota in naïve *Tipe^-/-^* mice are not altered compared with naïve WT mice. The 16S rRNA gene profiling data for fecal microbiome from 8-week-old naïve WT (n=5) and *Tipe^-/-^* (n=5) mice. **A** The relative abundances of the top 7 bacteria at the phylum level in mice were determined. **B** Analysis of α diversity (observed_species) in mice. The statistical analysis was performed from an independent experiment by Student’s t test (n.s. non-significant).


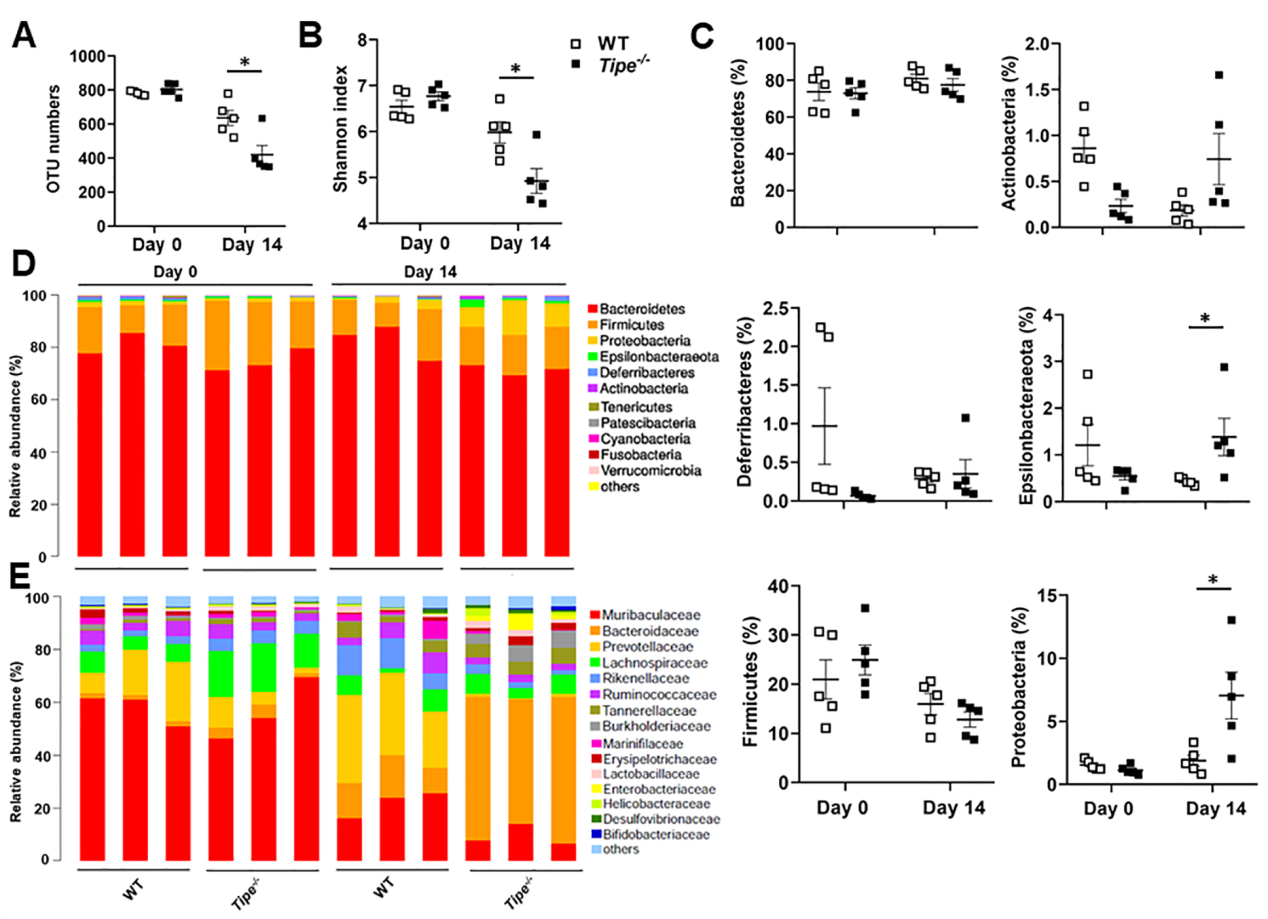


**Supplemental Figure 5.** Inflammation-associated microbiota are increased at early stages of CAC development. **A-B** Operational taxonomic unit (OTU) abundances (**A**) and Shannon-Wiener diversity index (Shannon index) (**B**) of the fecal specimens from WT (n=5) and *Tipe^-/-^* (n=5) mice on day 0 or day 14 of the CAC model by 16S rRNA sequencing. **C** Quantification analysis of phylum level microbiota distribution in the feces as in (**A**) by 16S rRNA sequencing. **D-E** Relative abundance of microbiota at the top 15 phylum level (**D**) or the top 15 family level (**E**) in the feces by taxon-based analysis as in (**A**) by 16S rRNA sequencing. Data are presented as mean± SEM and are representative of three independent experiments. Student’s t test (**A**, **B** and **C**), **p*<0.05.


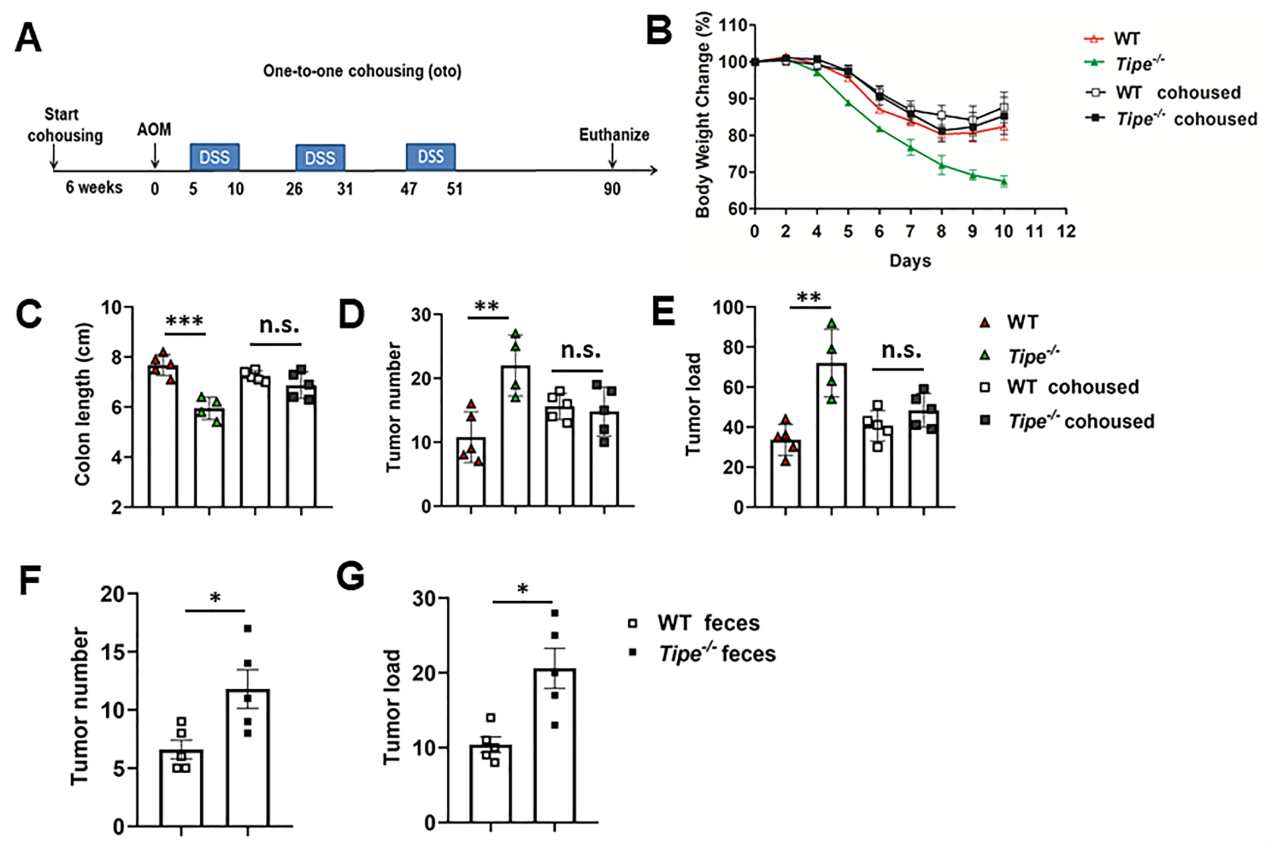


**Supplemental Figure 6.** Cohousing experiments of WT and *Tipe^-/-^* mice. **A** Schematic of cohousing strategy. WT and *Tipe^-/-^* mice were cohoused in a 1:1 ratio (oto) for 6 weeks. Mice were then administrated with DSS for the acute colitis model or AOM/DSS for the CAC model depending on the experiments. **B-C** For the acute colitis model, monohoused or cohoused WT and *Tipe^-/-^* mice were given 2.0% DSS in the drinking water for 5 days and then given regular drinking water until day 10. Body weight changes were assessed (**B**) and colon lengths were measured in each mouse on day 10 (**C**) (n=4 or 5 per group). **D-E** For the CAC model, monohoused or cohoused WT and *Tipe^-/-^* mice were subjected with AOM/DSS protocol. Both colon tumor number (**D**) and tumor load (**E**) of WT and *Tipe^-/-^* mice were determined on day 90 after AOM/DSS treatment (n=4 or 5 per group). **F-G** Recipient WT mice with antibiotic pretreatment and feces transfer were subjected to AOM/DSS protocol (n= 5 per group). Tumor number (**F**) and tumor load (**G**) were determined. Data are presented as mean± SEM and are representative of two independent experiments. Student’s t test (**B**, **C**, **D**, **E**, **F** and **G**), **p*<0.05,***p*<0.01, ****p*<0.001.

.


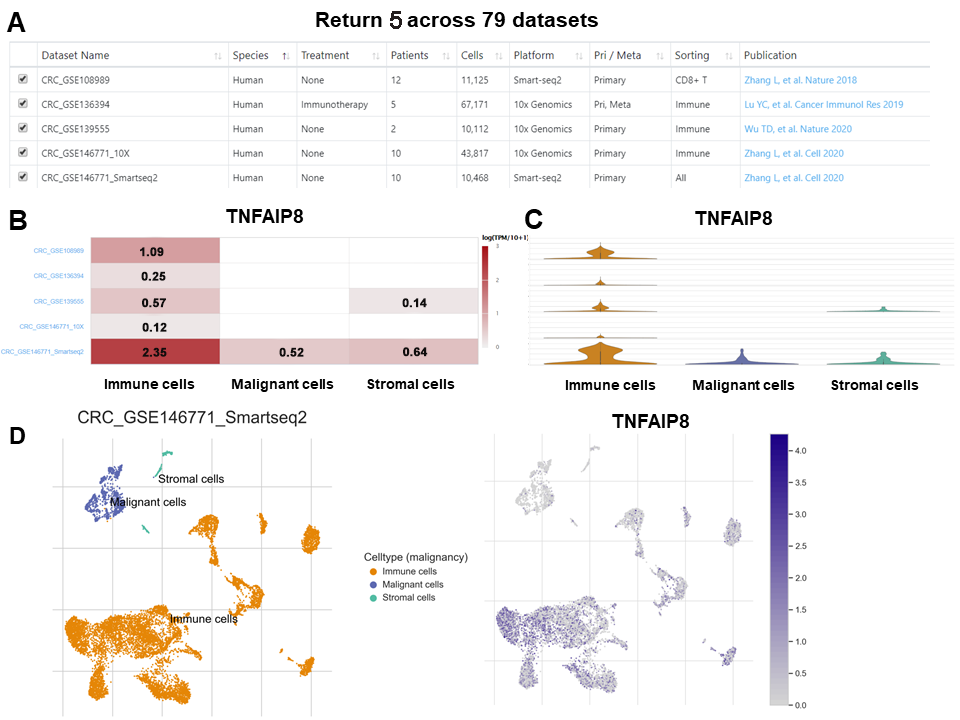


**Supplemental Figure 7.** Human TNFAIP8 gene module of Tumor Immune Single Cell Hub (TISCH). **A** Datasets on human CRC are indicated. **B-C** The heatmap (**B**) and violin plot (**C**) depicting the average expression of TNFAIP8 gene in different cell types, including immune cells and stromal cells across three mouse CRC datasets are shown. The color indicates the expression level of TNFAIP8 gene. The grid violin plot reflects the distribution of TNFAIP8 gene expression in different cell types across the datasets as indicated. **D** The overview tab of the selected CRC_GSE146771_Smartseq2 dataset. The expression of TNFAIP8 gene at single-cell and cell-type resolution is displayed in the CRC_GSE146771_Smartseq2 single-dataset module.
