# Supplementary material for: TNFAIP8 protein functions as a tumor suppressor in inflammation-associated colorectal tumorigenesis
Source: Cell Death Dis. 2022 Apr 6;13(4):311. doi: 10.1038/s41419-022-04769-x (PMC8986800; doi:10.1038/s41419-022-04769-x)

Uncropped blots for Figure 2C

p-P65

180 KD
130 KD
100 KD
70 KD
55 KD

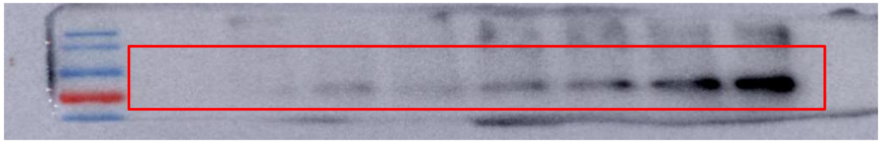

P65

180 KD
130 KD
100 KD
70 KD
55 KD

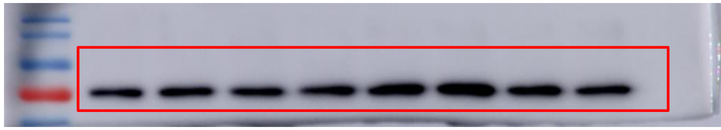

p-STAT3

180 KD
130 KD
100 KD
70 KD
55 KD

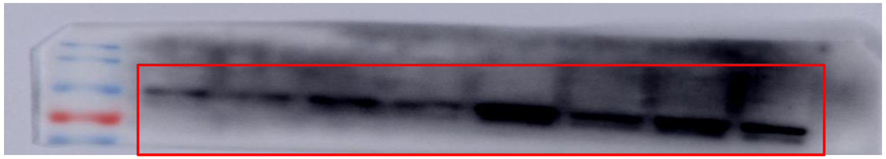

STAT3

180 KD
130 KD
100 KD
70 KD
55 KD

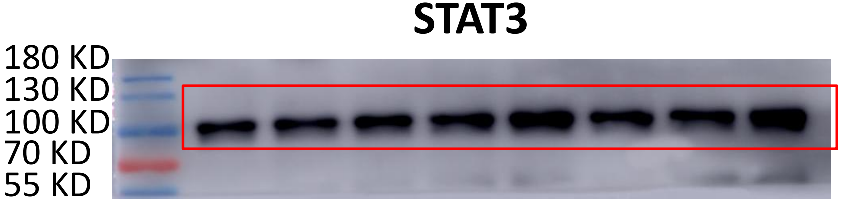

Actin

55 KD
40 KD
35 KD

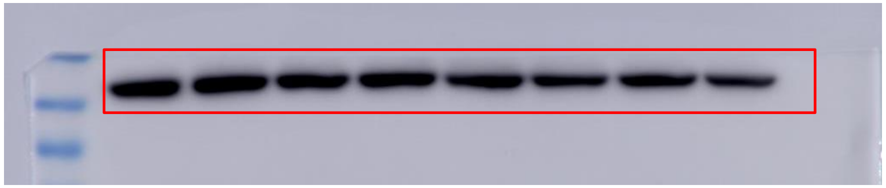

Uncropped blots for Figure 4F

P-Histone H2A.X

25 KD

15 KD

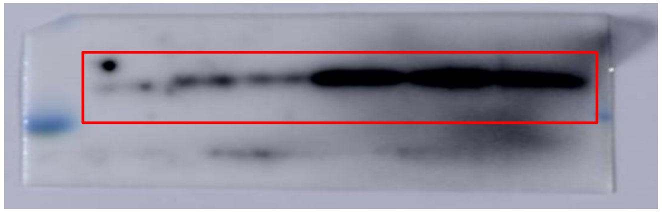

Histone H2A.X

25 KD

15 KD

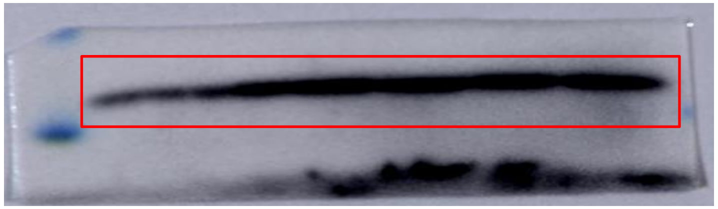

Actin

55 KD

40 KD

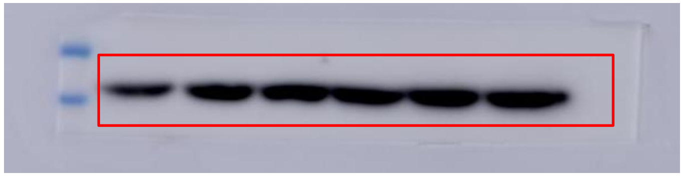

Supplement: Supplementary file 3 — Uncropped original western blots [file 41419_2022_4769_MOESM3_ESM.pdf]
